# Glucocorticoid Impaired the Wound Healing Ability of Endothelial Progenitor Cells by Reducing the Expression of CXCR4 in the PGE2 Pathway

**DOI:** 10.3389/fmed.2018.00276

**Published:** 2018-09-28

**Authors:** Erica Carolina, Toshiki Kato, Vuong Cat Khanh, Kana Moriguchi, Toshiharu Yamashita, Kosuke Takeuchi, Hiromi Hamada, Osamu Ohneda

**Affiliations:** ^1^Laboratory of Regenerative Medicine and Stem Cell Biology, Graduate School of Comprehensive Human Sciences, University of Tsukuba, Tsukuba, Japan; ^2^Ph.D. Program in Human Biology, School of Integrative Global Majors, University of Tsukuba, Tsukuba, Japan; ^3^Department of Obstetrics and Gynecology, Graduate School of Comprehensive Human Sciences, University of Tsukuba, Tsukuba, Japan

**Keywords:** endothelial progenitor cells, glucocorticoids, CXCR4, wound healing, prostaglandin E2, HIF2α

## Abstract

**Background:** Endothelial progenitor cells (EPCs) can be used to treat ischemic disease in cell-based therapy owing to their neovascularization potential. Glucocorticoids (GCs) have been widely used as strong anti-inflammatory reagents. However, despite their beneficial effects, side effects, such as impairing wound healing are commonly reported with GC-based therapy, and the effects of GC therapy on the wound healing function of EPCs are unclear.

**Methods:** In this study, we investigated how GC treatment affects the characteristics and wound healing function of EPCs.

**Results:** We found that GC treatment reduced the proliferative ability of EPCs. In addition, the expression of CXCR4 was dramatically impaired, which suppressed the migration of EPCs. A transplantation study in a flap mouse model revealed that GC-treated EPCs showed a poor homing ability to injured sites and a low activity for recruiting inflammatory cells, which led to wound healing dysfunction. Impairment of prostaglandin E2 (PGE2) synthases, cyclooxygenase (COX2) and microsomal PGE2 synthase 1 (mPEGS1) were identified as being involved in the GC-induced impairment of the CXCR4 expression in EPCs. Treatment with PGE2 rescued the expression of CXCR4 and restored the migration ability of GC-treated EPCs. In addition, the PGE2 signal that activated the PI3K/AKT pathway was identified to be involved in the regulation of CXCR4 in EPCs under the effects of GCs. In addition, similar negative effects of GCs were observed in EPCs under hypoxic conditions. Under hypoxic conditions, GCs independently impaired the PGE2 and HIF2α pathways, which downregulated the expression of CXCR4 in EPCs. Our findings highlighted the influences of GCs on the characteristics and functions of EPCs, suggesting that the use of EPCs for autologous cell transplantation in patients who have used GCs for a long time should be considered carefully.

## Introduction

Glucocorticoids (GCs) have been widely used as extremely effective agents for suppressing inflammation associated with various kinds of diseases (1). However, despite its beneficial effects, GC treatment is associated with several deleterious effects, including impaired wound healing (2). This side effect happens either via the transrepression of pro-inflammatory cytokines, growth factors, matrix proteins and matrix protease or via direct inhibitory influence on genes that are important for skin regeneration (3). GCs act as the synthetic analogs of human natural endogenous GCs, which exert their effects by binding to GC receptors (GRs), thereby regulating the expression of target genes that play important roles in the wound healing process (4). In addition, GCs also cause the dysfunction of cells involved in wound healing (5, 6). We recently demonstrated that GC treatment impairs the wound healing function of adipose tissue-derived mesenchymal stem cells (MSCs) via the downregulation of stromal cell-derived factor 1 (SDF-1) (5).

Wound healing is a complex process that requires the contribution of many cell types (7). Central to this, MSCs and endothelial progenitor cells (EPCs) play crucial roles in promoting wound healing (7). While MSCs contributes to the wound healing process mostly via paracrine effects, secreting a mixture of growth factors and vesicles to recruit numerous of cells to the wound sites and support these cells' functions, EPCs are the key effectors of neovascularization and are involved in the formation of blood vessels and maintenance of the function of the vascular endothelium (8). EPCs possess the self-renewal potential, differentiation ability to endothelial cells, and neovascularization capability toward new blood vessel formation (9, 10). A previous study showed that EPCs accelerate wound healing not only as the endothelial substrate of blood vessels but also by migrating to the injured tissue, exerting their effects by excreting proangiogenic factors (10).

The definition of an EPC has been controversial. Originally, EPCs were identified as the cell population isolated from human peripheral blood that expresses the CD34 and Flk1 markers (11). However, recently studies have highlighted that positive with CD34 and Flk1 are insufficient to define EPCs *in vitro* (8). Several studies have reported the two distinct types of EPCs, early and late EPCs, which early EPCs are characterized by the expression of CD45 and CD14, together with some endothelial cell (EC) markers, and have a short lifespan of 3–4 weeks. Additionally, late EPCs are characterized by EC markers, such as CD31, CD34, VEGFR2, and VE-cadherin, but are negative for myeloid markers (12). We previously reported a novel method for isolating EPCs according to the aldehyde dehydrogenase (ALDH) activity which showed that the expression of CD34 declines during the culturing of DiI-Ac-LDL–positive/CD45^−^/CD31^+^ cells; whereas, ALDH activity was retained stably in EPCs in long term culture (13). Of note, EPCs with a low ALDH activity (Alde-low) possess a higher migratory ability toward damaged tissue and show a better recover ability in ischemic wound healing than Alde-high EPCs (13). Under hypoxic conditions, Alde-low EPCs are highly responsive and show the upregulation of hypoxic condition inducible factor 2α (HIF2α), which regulates the expression of CXCR4, a major chemokine receptor for cell migration in response to SDF-1 (13). Therefore, Alde-low EPCs can be considered promising candidates for wound treatment (13).

Autologous transplantation of EPCs is considered a prospective approach for therapeutic revascularization and chronic wound (14). It is reported that autoimmune diseases and its drug-based therapy, such as GC, account for 20% of chronic wound cases (15). Therefore, the patients who have been receiving GC treatment are one of the major targets for EPC therapy. However, similar to other cell sources, the introduction of autologous EPCs transplantation into clinical treatment is still being met with difficulty due to the negative influence of patients' medical backgrounds on the outcome of the treatment (16, 17). Ensuring the EPCs' potential is crucial for achieving the best outcome of EPC-based therapy. A previous study suggested that chronic GC treatment reduces the number of circulating EPCs in the patients (16). However, no report has yet clarified the influences of GCs on the wound healing ability of EPCs. Thus, whether or not the outcomes of EPC-based therapy are worsened in patients who have been chronically treated by GCs remains unclear.

In the present study we examined whether or not GCs interfere with the wound healing ability of Alde-low EPCs. We found that GCs downregulated the expression of CXCR4 in a transplantation flap mouse model, which impaired the migration and wound healing ability of EPCs. Treatment with PGE2 upregulated the EP4 receptor and activated the PI3K/AKT signaling which were involved in rescuing the detrimental effects of GC on the CXCR4 expression of EPCs. In addition, similar detrimental effects of GCs on the PGE2/CXCR4 pathway were noted in EPCs under hypoxic conditions. Of note, under hypoxic conditions, independent with the PEG2 pathway, the HIF2α pathway was also involved in the GC-impaired CXCR4 expression in EPCs. Taken together, these findings highlight the negative effects of GCs on the EPC functions, suggesting that autologous EPC therapy for patients receiving GC treatment be considered carefully.

## Materials and methods

### Isolation of umbilical cord blood-derived EPCs

All experiments involving human subjects were performed in accordance with the Guidelines for Medical and Health Research Involving Human Subjects, Ministry of Education, Culture, Sports, Science and Technology, Japan and the permission of the Institutional Ethics Review Committee of the University of Tsukuba. Human EPCs were isolated from umbilical cord blood (UCB), as previously described (13). Isolated EPCs were sorted based on the ALDH activity using ALDEFLUOR® system reagents (StemCell Technologies, Vancouver, Canada). The EPCs with the low ALDH activity (Alde-low EPCs) were used for further experiments in the present study. Alde-low EPCs were cultured with maintenance medium (Iscove's modified Dulbecco medium-IMDM, Invitrogen, Carlsbad, CA, USA)/10% FBS/5 ng/mL bFGF (PeproTech, NJ, USA), and 0.1% (v/v) penicillin-streptomycin (100 U/mL penicillin, 0.1 mg/mL streptomycin; Invitrogen) and incubated at 37°C in 5% CO_2_.

### A fluorescence activated cell sorting (FACS) analysis of EPCs

EPCs were harvested with trypsin, counted the cell number and a number of 2 × 10^5^ cells were reconstituted in 100 μl 2% FBS-containing PBS. For staining purpose, cells were incubated with the desired antibodies for 30 min at 4°C.

The following antibodies were utilized with the volume of 5μl as the recommendation of the manufacturer: PE-labeled anti-CD105 (323206, BioLegend), PE-labeled anti-CD73 (550257, BD Pharmingen), PE-labeled anti-CD31 (303106, BioLegend), allophycocyanine (APC)–labeled anti-CD45 (555485, BD Biosciences, San Jose, CA), and FITC-labeled anti-CD34 (555821, BD Biosciences). Purified-anti-VEGFR-2 were used as previously reported (13). APC-labeled anti-IgG1 (555751, BD Biosciences), PE-labeled anti-IgG1 (555749, BD Biosciences), FITC-labeled anti-IgG1 (555748, BD Biosciences) were used as the isotype controls. After staining, cells were washed by 2% FBS-containing PBS, centrifuged at 1,800 RPM for 3 min at 4°C, and reconstituted in 300 μl 2% FBS-containing PBS. A flow cytometer (MoFlo XDP; Beckman Coulter, Pasadena, CA, USA) collected 10,000 events for each group, and the isotype control IGg was used as the negative control.

In order to quantify CXCR4 expression in EPCs, the cells were stained with 5μl of PE-labeled anti-CXCR4 (306506, BioLegend) and performed the FACS analysis with the similar above protocol. PE-labeled anti-IgG1 (555749, BD Biosciences) was used as the isotype controls. The threshold for CXCR4-positivity was quantified by means of fluorescent intensity value subtracted with isotype control value IgG of each group.

### Cell proliferation assay

The proliferation of EPCs was evaluated by the growth curve and proliferation assay staining with Cell Counting Kit-8 (CCK-8, Dojindo, Tokyo, Japan).

For growth curve, EPCs were seeded at a number of 4 × 10^3^ cells/well in the 24 well-plate dishes and cultured at 37°C in 5% CO_2_ for 9 days. The culture medium was changed every 3 days. The cells were washed with sterile PBS and treated with 0.05% trypsin/EDTA (Invitrogen) at 24-h intervals for 9 days to separate single cells. Dead cells were excluded using trypan-blue staining solution (35525-02; Nacalai Tesque, Kyoto, Japan), and the numbers of live cells in triplicate dishes were counted using a hemocytometer.

For proliferation assay staining with CCK-8, EPCs were seeded at a number of 2.5 × 10^4^, 1.25 × 10^4^, and 6.25 × 10^3^ cells/well in 96-well microplate. Cells were incubated for 48 h and 10 μl of Cell Counting Kit-8 reagent was added to each well, then incubate for 2 h. The number of live cells were measured by colorimetric reading using a microplate reader (Varioskan, ThermoFisher Scientific, Massachusetts, USA) for 450 nm.

### Annexin-V/7AAD staining assay

EPCs were trypsinized and centrifuged at 1,000 RPM for 3 min at 4°C. Cells were then resuspended in 100 μl of 2% FBS and stained with 5 μl of PE annexin V and 7-AAD (BD BioSciences (CA, USA), and then incubated at 4°C in the dark for 30 min. FBS (2%, 300 μl) was added, and the apoptotic cells were analyzed by flow cytometry.

### *In vitro* migration assay

*Scratch assay:* An *in vitro* migration assay was performed as previously reported with minor modifications (18). EPCs with a number of 1.5 × 10^5^ cells were seeded onto 4-well plates with maintenance medium until they reached confluency after 24 h. Scratch wounds ~1 mm wide were created. After gentle washing of the detached cells with PBS, the growth medium was changed to 2% FBS-containing IMDM. The pictures of wound closure were taken every 6 h during 24-h post-scratching at 100 × magnification (10 × objective and 10 × eyepiece) under a microscope (Olympus, Tokyo, Japan). The cell migration was calculated using the ImageJ software program (NIH, MD, USA). The wound closure distance was measured at the beginning (T0) and end of the experiment (Tx). The following formula was used to convert the migrated area to percentage: Percentage (%) of wound closure = *T*_0_ – *T*_*X*_ = [1 – *X*/*T*_0_ × 100].

### Transwell migration assay

A volume of 600 μl SDF-1 (100 ng/ml) in IMDM medium was placed in the lower chamber of each well of 24 well-plates. Afterwards, a volume of 200 μl IMDM medium containing 3 × 10^4^ EPCs added in the upper chamber of 8 μm pore sized of filter membrane. After 24 h of incubation, the lower side of filter was washed with PBS and fixed with 2% PFA. The number of migrated EPCs which migrate toward SDF-1 was determined by Hematoxylin & Eosin staining and counted under the observation using a microscope (Olympus) at 100 × magnification.

### Gene expression analyses

Total RNA was isolated from cultured EPCs using extraction reagent (Sepasol-RNA I Super G; Nacalai Tesque), and reverse transcription was performed with 1 μg of the total RNA using a reverse transcription polymerase chain reaction (RT-PCR) kit (Toyobo, Osaka, Japan). The expression of the target genes was analyzed using a GeneAmp PCR System (Life Technologies, CA, USA) with Thunderbird SYBR qPCR Mix (Toyobo). Experiments were carried out in triplicate, and the expression of the target genes was calculated using the 2^−ΔΔ*CT*^ method. β-Actin was used as an internal control. The primer sequences used for quantitative RT-PCR are shown in Table 1.

**Table 1:** The primer sets for the quantitative polymerase chain reactions.

**Gene**	**Primer**	**Sequence**
βactin	Forward	GTGCGTGACATTAAGGAGAAGCTGTGC
	Reverse	GTACTTGCGCTCAGGAGGAGCAATGAT
VEGF	Forward	AGATGAGCTTCCTACAGCACAAC
	Reverse	AGGACTTATACCGGGATTTCTTG
PDGF-AA	Forward	TAGGGAGTGAGGATTCTTTGGACACCA
	Reverse	CAAATGCTCCTCTAACCTCACCTGGAC
FGF	Forward	AGAGCGACCCTCACATCAAGCTACAAC
	Reverse	ATAGCTTTCTGCCCAGGTCCTGTTTTG
Ang-1	Forward	GCCTGATCTTACACGGTGCT
	Reverse	GGCCACAAGCATCAAACCAC
SDF-1	Forward	AGAGCCAACGTCAAGCATCT
	Reverse	CTTTAGCTTCGGGTCAATGC
CXCR4	Forward	CCTTATCCTGCCTGGTTATTGTC
	Reverse	AGGATGAGGATGACTGTGGTCT
CXCR7	Forward	AGAGCTCACAGTTGTTGCAAAGTGC
	Reverse	GGTTCAAGATGTAGCAGTGCGTGTC
CCL2	Forward	GAATCACCAGCAGCAAGTGT
	Reverse	GTTTGGGTTTGCTTGTCCAGG
TGF-β	Forward	AGAGCTCCGAGAAGCGGTACCTGAACCC
	Reverse	GTTGATGTCCACTTGCAGTGTGTTATCC
COX-2	Forward	GGCAGGAGGTCTTTGGTCT
	Reverse	AACTGCTCATCACCCCATTC
mPGES-1	Forward	GGAACGACATGGAGACCATGTAC
	Reverse	TCCAGGCGACAAAAGGGTTA
EP-4	Forward	CCTCAGCGACTTTCGGCG
	Reverse	ACGAATACTCGCACACGAG

### *In vivo* wound healing model

C57BL/6 mice were purchased from Charles River Japan, Inc. (Kanagawa, Japan). All experiments were performed in compliance with the Fundamental Guidelines for Proper Conduct of Animal Experiment and Related Activities in Academic Research Institutions under the jurisdiction of the Ministry of Education, Culture, Sports, Science and Technology, Japan. Each experiment was repeated triplicate. In order to compare the wound healing ability of untreated EPCs and GC-treated EPCs, total 15 male mice at 12th week of age were used in each experiment and divided into three groups (5 mice/group): PBS, EPCs, GC-treated EPC. In order to examine the role of CXCR4 in the wound healing ability of EPCs, total nine male mice at 12th week of age were used in each experiment and divided into three groups (3 mice/group): untreated EPCs, GC-treated EPCs, and CXCR4 antibody-treated EPCs (AMD3100, Sigma-Aldrich). 100 nM of AMD3100 at 24-h treatment was used to block the CXCR4. The mice were anesthetized using avertin, and a skin incision (3 × 2 cm) was made to create an ischemia gradient as previously described (19). EPCs were treated with 100 nM GC for 24 h prior to surgery then labeled with PKH26 (Sigma-Aldrich, Missouri, USA) as the instruction of the manufacturer before transplantation. PBS and PKH26-labeled untreated EPCs were used as the controls. The EPCs were injected at a number of 5 × 10^5^/mouse through the tail vein. Skin tissue samples were fixed overnight with 4% paraformaldehyde; then, washed with PBS, soaked in sucrose 10% for 2 h, and finally in sucrose 20% overnight. Then, the frozen blocks of samples were made by embedded the samples in the O.C.T compound (Sakura Finetek, Tokyo, Japan) and freeze in liquid nitrogen. After that the tissues were sectioned before immunostaining. For observation, the flap tissues were collected at two different time points: on the third day of transplantation to analyze the inflammatory cell recruitment and on the seventh day of transplantation to analyze the neovascularization. Images of the ischemic flaps were captured on the seventh day of transplantation, and the necrotic areas were quantified using the Image J software program (NIH).

### Histological analyses

The frozen flap tissue sections were mounted, stained with hematoxylin and eosin (Wako, Osaka, Japan) and observed under a microscope (Olympus, Tokyo Japan). The inflammatory cells recruited in the ischemic area were visualized by immunohistochemical staining with rat anti-mouse CD45 (553078, BD Pharmigen), rat anti-mouse Mac1 (rat anti-mouse CD11b 550282, BD Pharmigen). The neovascularization was analyzed by immunohistochemical staining with rat anti-mouse CD31 (553370, BD Pharmigen) as the instruction of the manufacturer. Briefly, sections were washed in PBS and then rinsed in 10% H_2_O_2_ in PBS before blocking with blocking buffer containing Phosphate-Buffered Saline with 0.5% Tween 20 (PBST) and normal rabbit serum at a dilution of 4:1 (v/v) for 1 h at room temperature. After that, the sections were incubated with appropriate antibody overnight. On the following day, the sections were washed with PBST before incubating in the secondary antibody (POD Conjugate Anti-rat, MK-201, Takara, Shiga, Japan) for 30 min at room temperature. The sections were then washed with PBS and covered with 0.05% 3,3′-Diaminobenzidine (DAB) solution (Sigma-Aldrich) in PBS with H_2_O_2_ for 3 min then stopped detection by water. Quantification of the number of positive cells was performed at 200 × magnification (20 × objective and 10 × eyepiece) under a microscope (Olympus) by counting the brown dots in 10 fields.

### Western blotting

Cultured EPCs were harvested, counted and a number of 10^6^ cells were suspended in low-salt buffer (10 mM HEPES, 10 mM KCL, 1 mM dithiothreitol, 1 mM EDTA, protease inhibitor cocktail (PIC), and 1% Nonidet P-40, Roche Diagnostics, Basel, Switzerland), and nuclear pellets were collected by centrifugation. The nuclear pellets were then suspended in high-salt buffer (20 mM HEPES, 400 mM NaCl, 1 mM dithiothreitol, and 1 mM EDTA, PIC), and the nuclear extract was obtained. The total protein concentration was measured by Bradford assay (Bio-rad, CA, USA) as the instruction of the manufacturer. 30μg protein of each nuclear extract samples were separated on 10% sodium dodecyl sulfate-polyacrylamide gel electrophoresis (SDS-PAGE) and transferred onto a PVDF membrane (EMD Millipore, Darmstadt, Germany).

An immunoblotting analysis was performed. Briefly, the membranes were blocked with 5% Bovine Serum Albumin (Sigma-Aldrich, Missouri, USA) in Tris-Buffered Saline with 0.5% Tween 20 (TBST) for an hour at room temperature, then incubated incubation with the appropriate primary antibody overnight. Primary antibodies were diluted at the dilution of 1:1,000 (v/v) as the instruction of the manufacturer, including: Rabbit anti-human AKT (9272S, Cell Signaling Technology, Massachusetts, USA, phosphorylated AKT antibody (9271S, Cell Signaling Technology), rabbit anti-human HIF-2α antibody (NB100-122, Novus Biologicals, CO, USA), and goat anti-Lamin B antibody (M-20, Santa Cruz Biotechnology, Inc., CA, USA. The membranes were washed with TBST buffer then incubated with secondary antibodies, including: Horseradish peroxidase (HRP)-conjugated goat anti-rabbit IgG (32460, ThermoFisher Scientific) and rabbit anti-goat IgG (31402, ThermoFisher Scientific) with the dilution of 1:10,000 (v/v) for an hour at room temperature. Then, the membranes were washed and the enhanced chemiluminescence (GE Healthcare, Illinois, USA) was used for detection by a luminescent image analyzer (ImageQuant LAS4000, GE Healthcare). The band densities of target proteins were analyzed by ImageJ software (NIH). The relative expressions of target proteins were normalized with the total-LaminB expression. For the phosphorylation analysis, after incubation with phosphorylated antibody and detected the phosphorylated protein, the membranes were striped with Restore Stripping Buffer (ThermoFisher Scientific) for 15 min at room temperature, then, continued to incubate with rabbit Akt antibody. The Akt signals were detected and the membranes were then stripped and re-probed with LaminB antibody.

### Statistical analyses

Data were statistically analyzed using Mann Whitney U test using the GraphPad Prism 5 software program (GraphPad Software, CA, USA) as appropriate. Data are presented as the mean ± standard deviation. *P* < 0.05 was considered as significant.

## Results

### GC treatment impaired the proliferation ability of EPCs

**Figure 1:**
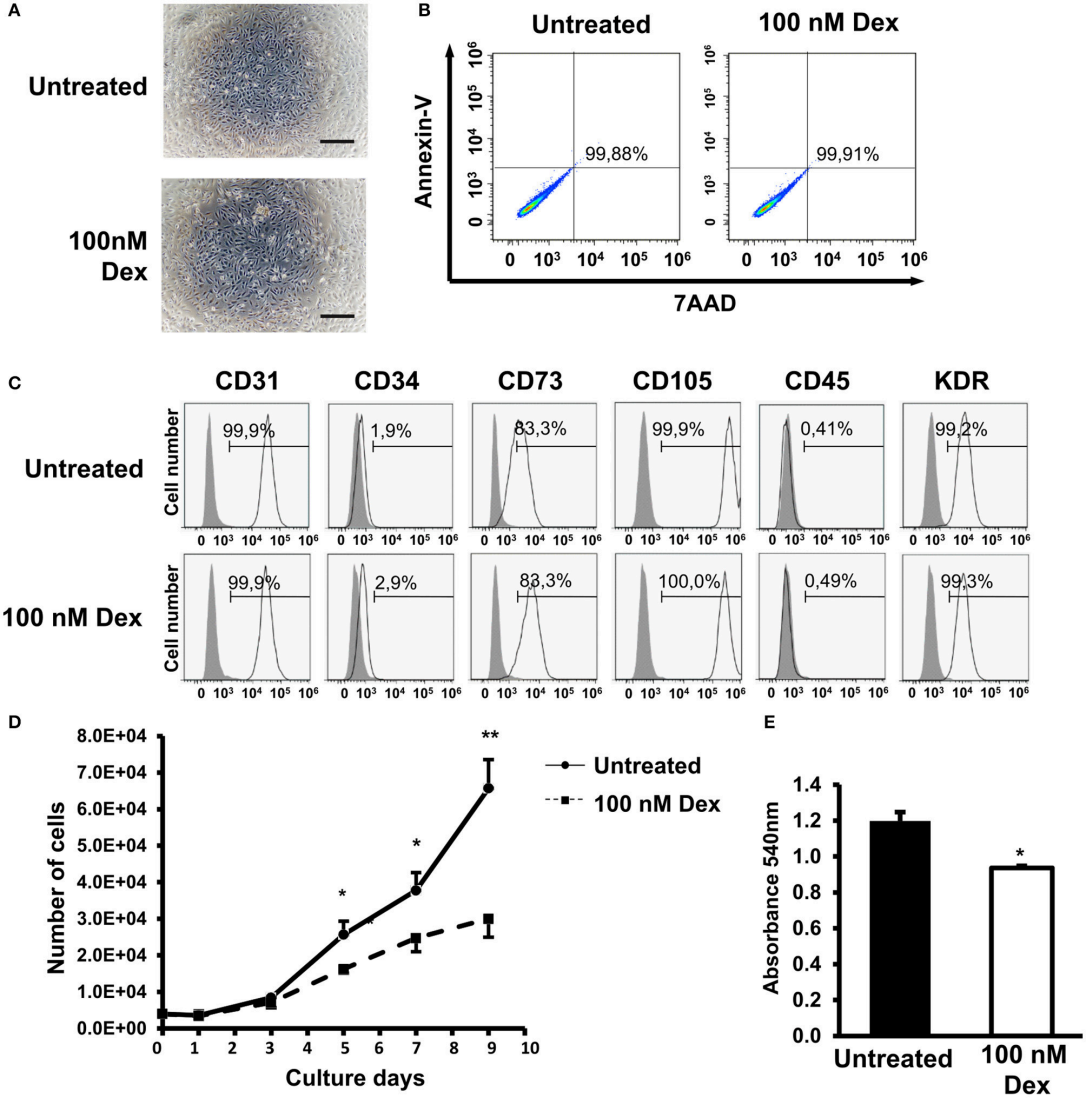
Glucocorticoid (GC) treatment impaired the proliferation ability of EPCs. **(A)** Cell morphology of untreated and GC-treated EPCs from one representative experiment. **(B)** Apoptotic cells of untreated and GC-treated EPCs. Cells were stained by Annexin V staining, then analyzed by the FACS analysis. The histograms were from one representative experiment. **(C)** Cell surface markers of untreated and GC-treated EPCs analyzed by FACS analysis. The histograms were from one representative experiment. **(D)** The growth curve of untreated and GC-treated EPCs from one representative experiment. **(E)** The proliferation assay of untreated and GC-treated EPCs examined by Cell counting kit-8. The chart shows the mean data from all experiments. Dexamethasone (100 nM, 24-h treatment) was used as a GC. Untreated: untreated EPCs, 100 nM Dex: GC-treated EPCs. The data represent the mean ± SD. *n* = 3, **P < 0.01, *P < 0.05. The scale bar indicates 100 μm. The experiment was repeated triplicate.

A previous report stated that GCs induced apoptosis in lymphocytes, demonstrating the negative effects of GCs on cells (20). However, thus far, no report has described the effects of GCs on EPCs; therefore, in the present study, we examined the characteristics of EPCs, including the cell morphology, viability, surface markers, and proliferation, under treatment with 100 nM dexamethasone as a GC. We found that GC treatment exerted no effects on the adherence or visual appearance of EPCs (Figure 1A). In addition, Annexin-V/7AAD staining assay data showed that 24-h treatment of GC showed no apoptosis induction or any cytotoxicity in EPCs (Figure 1B).

To examine the effects of GC on the EPC surface markers, a FACS analysis was performed. The data showed that there were no significant changes in the self-surface markers of EPCs under GC treatment, with both control and treated groups expressing high levels of CD31, CD34, CD105, CD73, and VEGFR2 and not expressing CD45 hematopoietic marker at all (Figure 1C). Next, we investigated the effects of GC treatment on the proliferation ability of EPCs. The data showed that the proliferation ability of EPCs treated with GC was significantly impaired compared to the untreated EPCs (Figures 1D,E). Taken together, these data show that GC impaired the proliferation ability of EPCs.

### GC impaired the *in vitro* migration ability of EPCs via the downregulation of CXCR4

**Figure 2:**
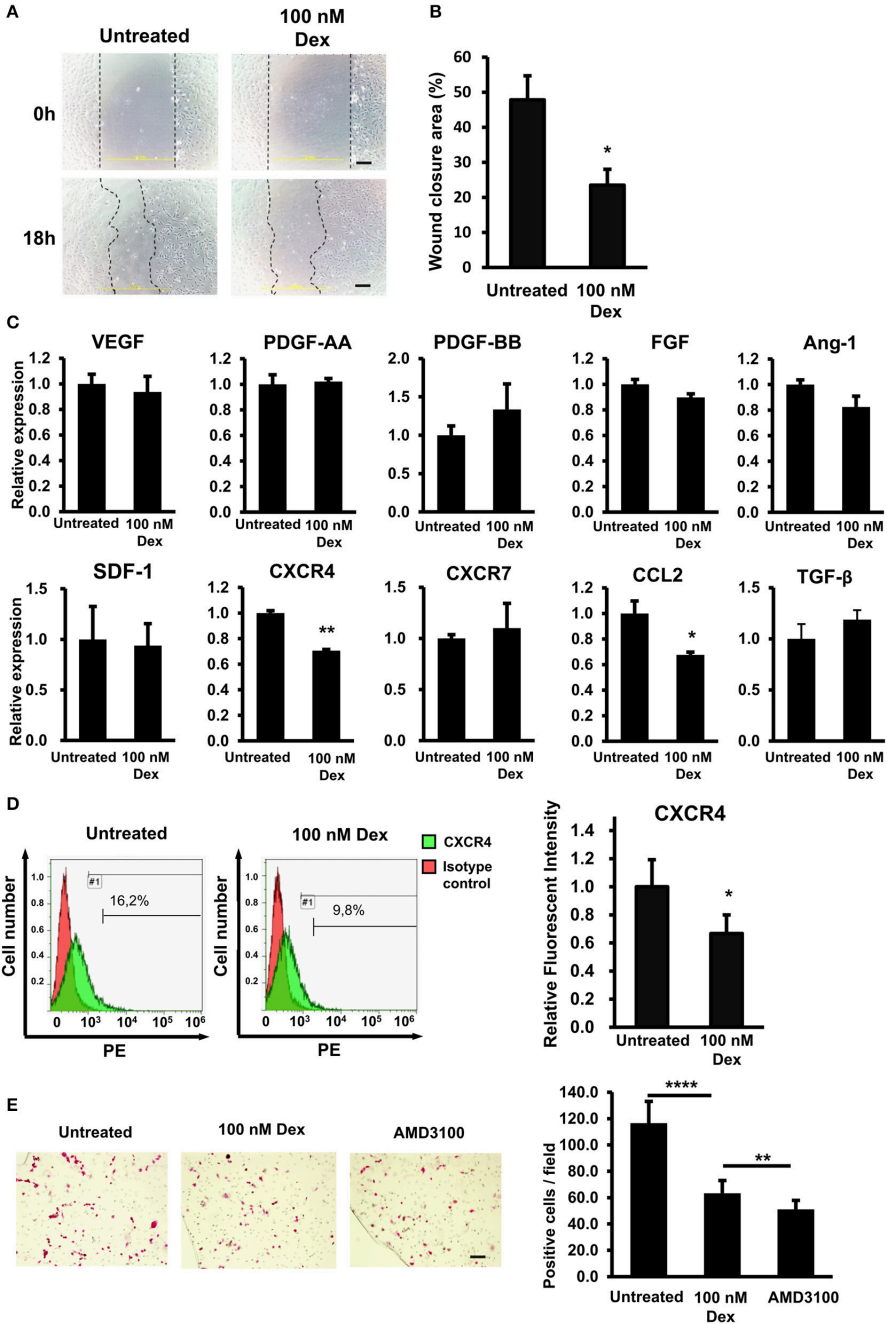
GCs impaired the *in vitro* migration ability of EPCs via the downregulation of CXCR4. **(A)** The *in vitro* wound healing ability of untreated and GC-treated EPCs by a scratch assay. The images showed the wound closures from one representative experiment taken under a microscope at 100× magnification. **(B)** Quantification of the wound closure area in a scratch assay of untreated and GC-treated EPCs. The data were displayed as the means from all experiments. **(C)** The relative mRNA expression of wound healing genes in untreated and GC-treated EPCs. The chart shows the mean data from all experiments. **(D)** The relative protein expression of CXCR4 in untreated and GC-treated EPCs identified by a FACS analysis. The FACS histograms were from one representative experiment. The chart shows the quantification of relative expression of CXCR4 protein. The data were displayed as the means from all experiments. **(E)** The transwell assay of untreated, GC-treated EPCs, and CXCR4 antibody-treated EPCs toward SDF-1. AMD3100 (100 nM, 24-h treatment) was used as the CXCR4 antibody. The images were the migrated EPCs from one representative experiment taken under a microscope at ×100 magnification. The chart shows the quantification of number of migrated EPCs. The data were displayed as the means calculated from all experiments. Dexamethasone (100 nM, 24-h treatment) was used as a GC. Untreated: untreated EPCs, 100 nM: GC-treated EPCs. The data represent the mean ± SD. *n* = 3, ****P < 0.0001, **P < 0.01, *P < 0.05. The scale bar indicates 100 μm. The experiment was repeated triplicate.

Previous studies have described the negative effects of GCs on the wound healing process (3, 20). Recently, we also reported that GCs impair the wound healing function of mesenchymal stem cell by reducing the expression of SDF-1 (5). Therefore, in the present study, we thought that GCs might also affect the wound healing function of EPCs. To test this hypothesis, we evaluated the wound healing function of EPCs under GC treatment. First, because the EPC migration ability is critical for their biological properties, the effect of GC on the migration ability of EPCs was evaluated by a scratch assay as an *in vitro* wound healing model (21). As shown in Figure 2A, GC-treated EPCs showed a lower capability of wound closure than the control, indicating a reduced migration ability of EPCs on exposure to GC (Figures 2A,B).

Next, we assessed the expression profiles of the wound healing-related genes in EPCs. Numerous reports have shown that EPCs express multiple chemokines and their receptors related to mobilization (SDF-1, CXCR4, CXCR7), angiogenesis (Ang-1, TGFβ), immunomodulatory (CCL2), and growth factors (PDGF, VEGF, FGF) that play a critical role in wound healing (22, 23). To clarify how GC interferes with the wound healing capacity of EPCs, we examined the mRNA expression of those genes in GC-treated EPCs.

We observed a significantly impaired expression of CXCR4, which related to the mobility (24) and CCL2, which related to the inflammatory cell recruitment (25, 26) in GC-treated EPCs compared to that in control cells (Figure 2C). In addition, several genes that contribute to EPC migration, such as the extracellular matrix (ECM) protease MMP9 and homing and adherence factor VCAM 1, were also downregulated due to GC treatment (data not shown). Consistent with the mRNA expression data, a further FACS analysis showed a reduction in the CXCR4 surface protein level (Figure 2D).

It is reported that the SDF-1/CXCR4 cascade is responsible for the mobilization of EPCs (10). Therefore, we next performed the transwell assay to examine the effects of GC treatment on the migration ability of EPCs toward the signal of SDF-1. As expected, GC-treated EPCs showed the low migration ability toward SDF-1 compared to the untreated cells (Figure 2E). In addition, treatment EPCs with a CXCR4 antibody, AMD3100, also showed the similar impaired migration ability, suggesting the direct role of CXCR4 that regulates the mobility of EPCs (Figure 2E). Taken together, these results showed that GC impaired wound healing ability *in vitro* and the migration ability toward SDF-1 of EPCs by the downregulation of CXCR4 and CCL2.

### GC impaired the wound healing ability of EPCs

**Figure 3:**
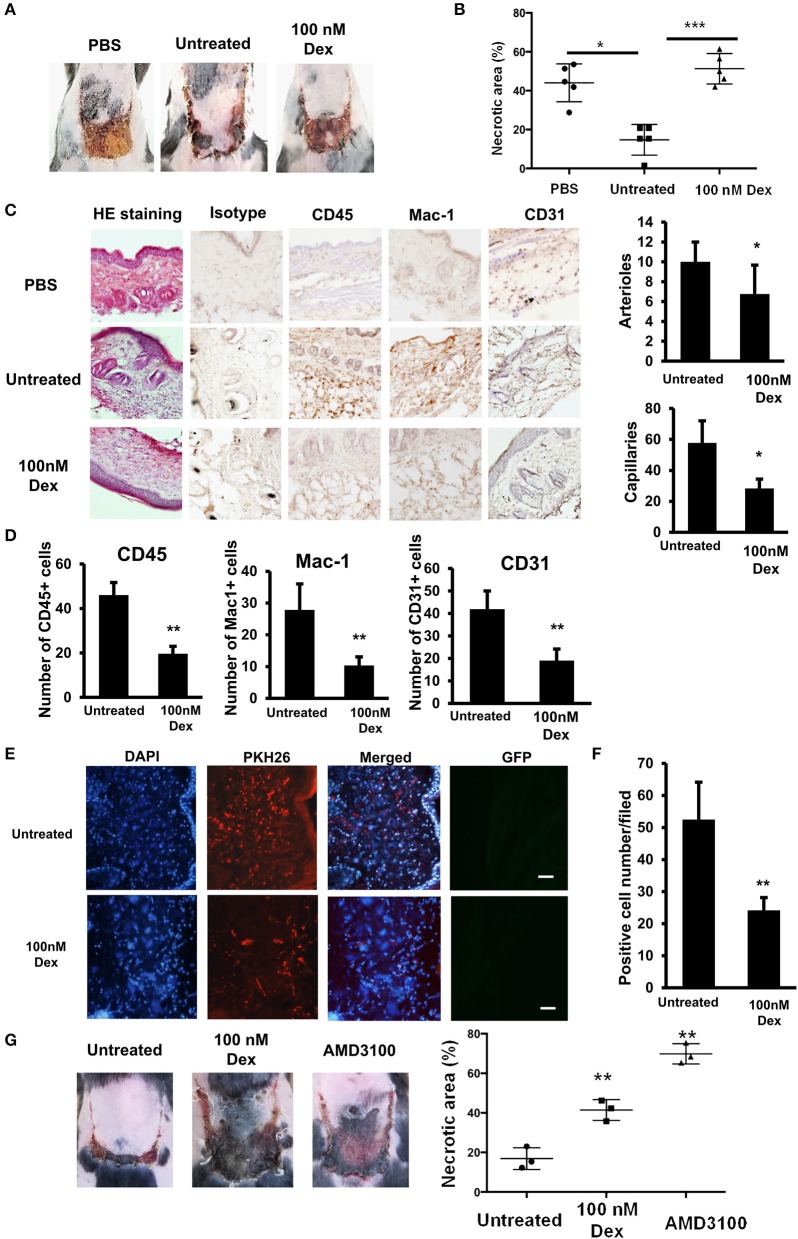
GCs impaired the wound healing ability of EPCs. **(A)** Wound healing ability of untreated and GC-treated EPCs in flap mouse model. **(B)** The necrotic area of the flap tissues injected with untreated and GC-treated EPCs. **(C)** Immunohistochemistry of CD45-, MAC-1- (at day 3 post-transplantation), and CD31-positive cells (at day 7 post-transplantation) in flap tissues injected with untreated and GC-treated EPCs observing under a microscope at 200× magnification. **(D)** Quantification of CD45-, MAC-1- (at day 3 post-transplantation), and CD31-positive cell numbers (at day 7 post-transplantation) in flap tissues injected with untreated and GC-treated EPCs observing under a microscope at 200× magnification. **(E)** PKH-positive EPCs in flap tissues injected with untreated and GC-treated EPCs observing under a microscope at 200× magnification. **(F)** Quantification of PKH-positive EPCs in flap tissues injected with untreated and GC-treated EPCs observing under a microscope at 200× magnification. From **A–F**, the data were calculated from five individual mice from one representative experiment. The data represent the mean ± SD. *n* = 5, ***P < 0.001, **P < 0.01, *P < 0.05. **(G)** Wound healing ability of untreated and CXCR4 antibody-treated EPCs in flap mouse model. AMD3100 (100 nM, 24-h treatment) was used as a CXCR4 antibody. The data were calculated from three individual mice from one representative experiment. The data represent the mean ± SD. *n* = 3, **P < 0.01. Dexamethasone (100 nM, 24-h treatment) was used as a GC. Untreated: untreated EPCs, 100 nM: GC-treated EPCs. The scale bar indicates 100 μm. The experiment was repeated triplicate.

We next examined the effects of GC on the wound healing ability of EPCs by a transplantation study using an *in vivo* mouse flap model. The wound healing functions of GC-treated EPCs were analyzed and compared to those in untreated cells. We found that the injection of untreated EPCs healed the wounds of mice with a reduction in the necrotic areas at 7 days after transplantation. In contrast, the wound healing ability of the GC-treated EPCs was significantly impaired, with some necrotic areas remaining, similar to control mice without any cell transplantation (Figures 3A,B).

Because the GC-treated EPCs showed the impaired expression of CCL2 which related to the inflammatory cell recruitment ability (25, 26), we performed a histological analysis of the wound healing process on the third day after transplantation and the data revealed that mice transplanted with untreated EPCs showed an increased recruitment of CD45- and Mac-1 positive cells to the wound sites compared to mice transplanted with GC-treated EPCs (Figures 3C,D). In addition, on the seventh day after transplantation, mice injected with untreated EPCs showed a greater number of CD31-positive vascular endothelial cells than mice injected with GC-treated EPCs (Figures 3C,D). In addition, to examine the effects of GC treatment on the homing and migration ability of EPCs to the wound sites, we quantified the PKH-labeled EPCs in the wound regions on the first day of transplantation. We found that there were fewer PKH-positive cells at the wound sites of mice transplanted with GC-treated EPCs than in those injected with untreated EPCs (Figures 3E,F).

We previously reported the impaired wound healing ability of EPCs with high activity of ALDH (Alde-high EPCs) due to the low expression of CXCR4 compared to those with low activity of ALDH (Alde-low EPCs) (19). In order to clarify the direct role of CXCR4 in the wound healing ability of EPCs, we blocked the activity of CXCR4 by its antibody, AMD3100, and compared the necrotic area in the transplantation study. As expected, similar to the effects of GC, blocking CXCR4 in EPCs showed the impaired wound healing function in the flap mice (Figure 3G), suggesting CXCR4 is responsible for the wound healing ability of EPCs.

Taken together, these data demonstrated that GCs impaired the wound healing functions of EPCs.

### GC-impaired prostaglandin E2 production was involved in the downregulation of CXCR4 in EPCs

**Figure 4:**
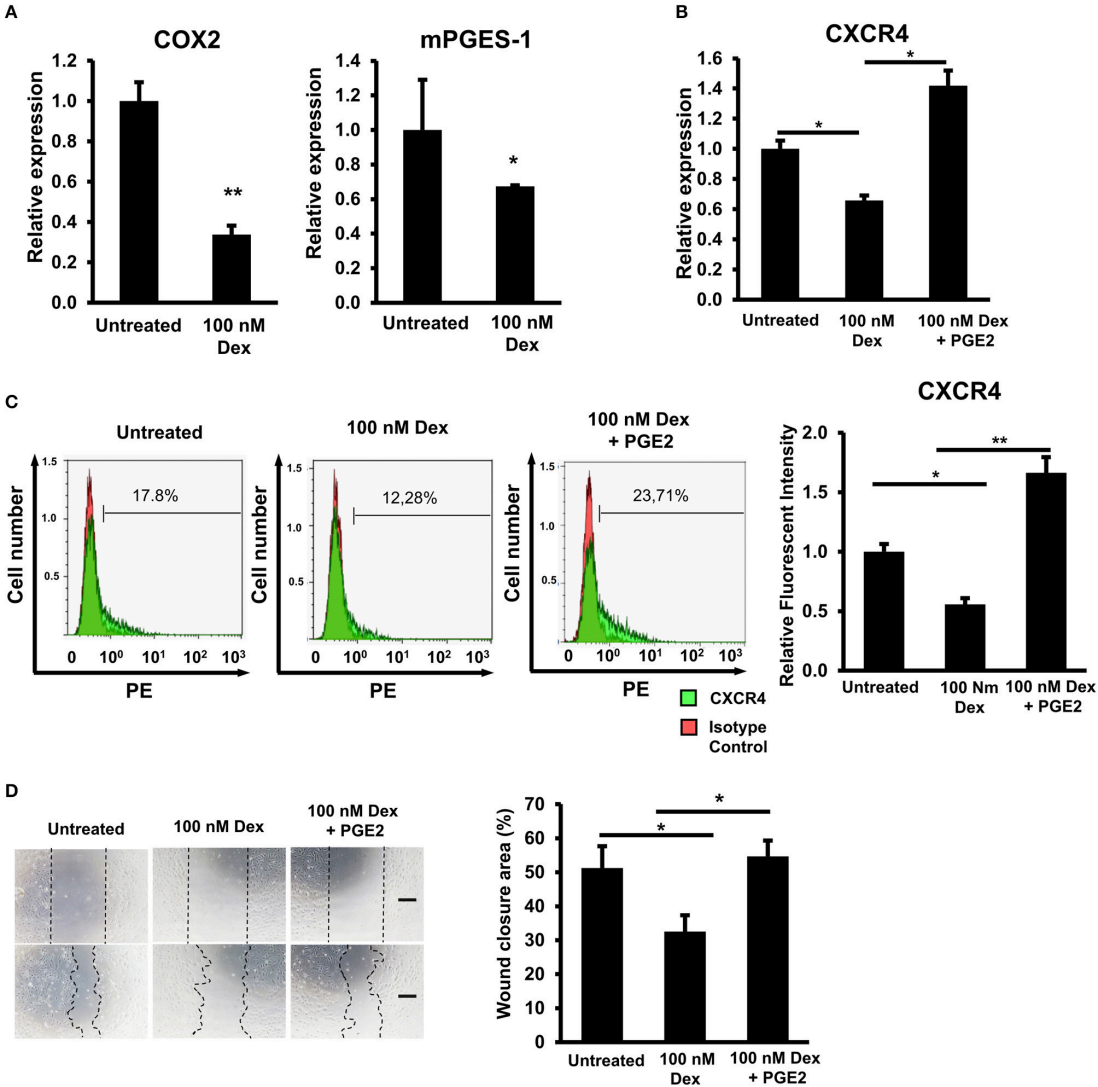
GC-impaired prostaglandin E2 production was involved in the downregulation of CXCR4 in EPCs. **(A)** The relative mRNA expression of COX2 and mPEGS1 in untreated and GC-treated EPCs. **(B)** The relative mRNA expression of CXCR4 in untreated, GC-treated EPCs, and GC-treated EPCs in the presence of PGE2. **(C)** The relative protein expression of CXCR4 in untreated, GC-treated EPCs, and GC-treated EPCs in the presence of PGE2 by a FACS analysis. **(D)** An *in vitro* scratch assay of untreated, GC-treated EPCs, and GC-treated EPCs in the presence of PGE2 observing under a microscope at 100× magnification. Dexamethasone (100 nM, 24-h treatment) was used as a GC. Untreated: untreated EPCs, 100 nM: GC-treated EPCs, 100 nM + PGE2: GC-treated EPCs in the presence of PGE2. The data represent the mean ± SD. *n* = 3, **P < 0.01, *P < 0.05. The scale bar indicates 100 μm. The experiment was repeated triplicate. The charts showed the overall mean data from all experiments.

Previous studies have shown that GCs are a strong inhibitor of the arachidonic acid cascade, resulting in less production of prostaglandin E2 (PGE2) in macrophages, vascular smooth muscle cells, and MSCs (27–30). In addition, other studies have suggested that the expression of CXCR4 is elevated in the presence of PGE2 (31). Therefore, we next investigated whether or not GC impaired the production of PGE2 to cause the downregulation of CXCR4 expression in EPCs. Cyclooxygenase (COX) and prostaglandin dehydrogenase (mPGES) are reported to be key enzymes in the catabolism of PGE2 (32–34). We found that GC treatment impaired the expression of COX-2 and mPGES-1 in EPCs, implying the negative effects of GCs on the PGE2 production of these cells (Figure 4A).

Next, in order to clarify the involvement of PGE2 in the downregulation of the CXCR4 expression in GC-treated EPCs, we examined whether or not treatment with PGE2 could reverse the expression of CXCR4 of these cells. The data showed that treatment of PGE2 rescued the GC-impaired mRNA and protein expression of CXCR4 in EPCs (Figures 4B,C). Furthermore, consistent with the upregulation of CXCR4 in the presence of PGE2, the migratory capacity of GC-treated EPCs was also recovered by PGE2 treatment (Figure 4D). Taken together, these results suggested that GC downregulated the expression of CXCR4 in EPCs by the impairment of PGE2 synthesis via COX-2 and mPGES-1.

### EP4/AKT signaling was involved in the GC-downregulated PGE2 in EPCs

**Figure 5:**
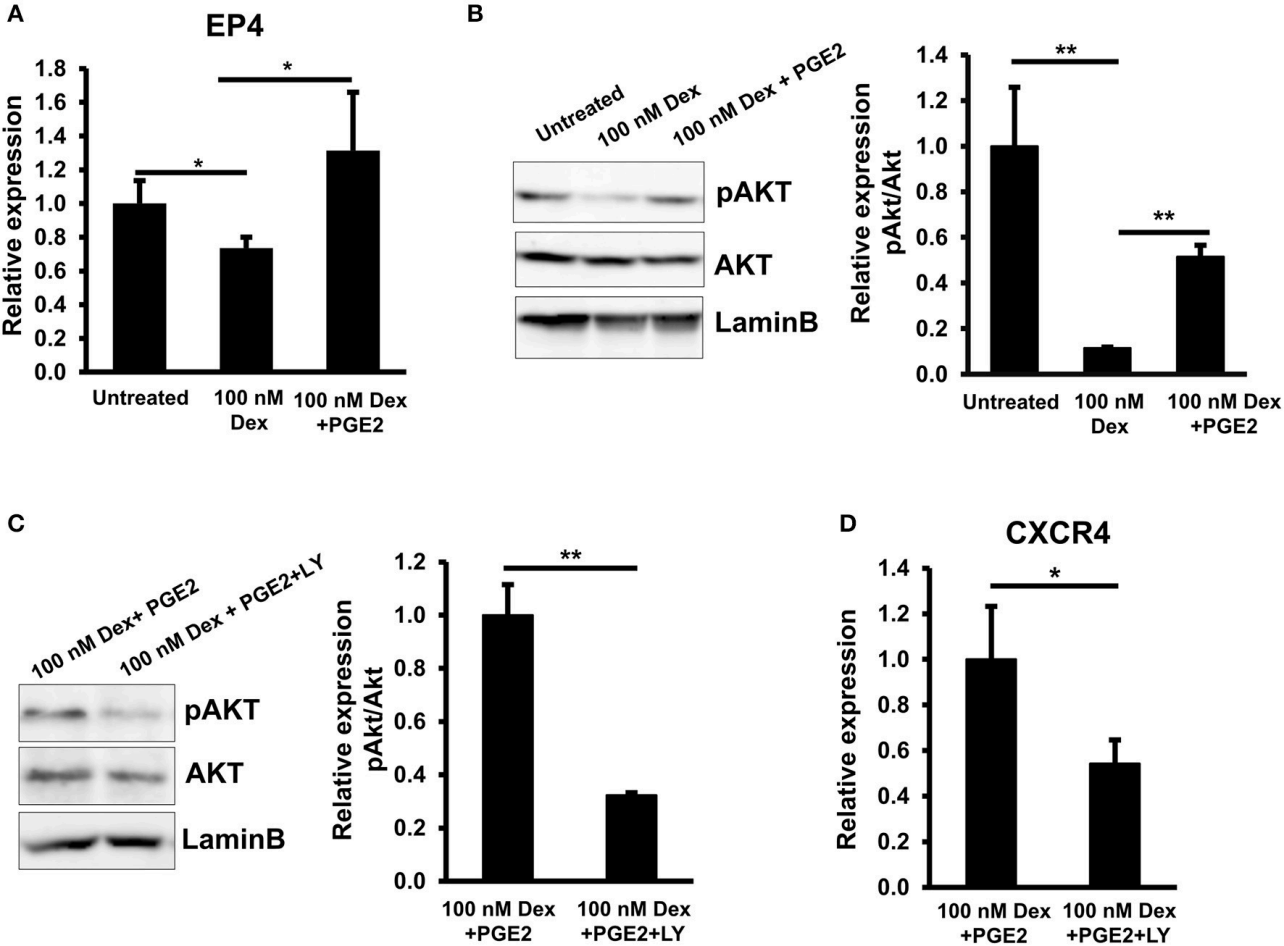
GCs downregulated CXCR4 via the impaired PGE2/EP4/AKT signaling in EPCs. **(A)** The relative mRNA expression of EP4 in untreated, GC-treated EPCs, and GC-treated EPCs in the presence of PGE2. **(B)** The relative protein expression of pAKT/AKT in untreated, GC-treated EPCs, and GC-treated EPCs in the presence of PGE2. **(C)** The inhibition of PI3K/AKT pathway in GC-treated EPCs in the presence of PGE2. LY294002 was used as a PI3K inhibitor. **(D)** The relative mRNA expression of CXCR4 in GC-treated EPCs in the presence of PGE2 and PI3K inhibitor. Dexamethasone (100 nM, 24-h treatment) was used as a GC. Untreated: untreated EPCs, 100 nM: GC-treated EPCs, 100 nM + PGE2: GC-treated EPCs in the presence of PGE2, 100 nM + PGE2 + LY: GC-treated EPCs in the presence of PGE2 and PI3K inhibitor. LY: LY294002. The data represent the mean ± SD. *n* = 3, **P < 0.01, *P < 0.05. The experiment was repeated triplicate. The charts showed the overall mean data from all experiments.

A previous reports showed that PGE2 exerted its activity by interaction with a G-protein-coupled receptor family (GPCRs) consisting of EP1, EP2, EP3, and EP4 subtypes, resulting in the different signal transduction and regulation of the expression of numerous target genes (35). To identify which PGE2 receptors are affected by GC, we analyzed the gene expression of these subtypes in EPCs in the presence of GC. We found that, among these four receptors, GC treatment only impaired the expression of EP4 in EPCs. In addition, treatment with PGE2 reversed the negative effects of GC on the EP4 expression, suggesting that EP4 is the PGE2 receptor related to the effects of GC on EPCs (Figure 5A).

The EP2 and EP4 receptors which mediate the increased cAMP concentrations had been thought to have similar effects in some biological process; however, recently, their distinct roles were reported (36, 37). It might be because of the selective expression of either of them in cells and the selective actions on the different signaling pathways (37). It is reported that EP4 but not EP2 couples to PI3K which regulates the migration of dendritic cells in the mouse suggesting the role of EP4/PI3K/AKT in the regulation of migration ability of the cells (37). Therefore, we next examined the role of PI3K/AKT pathways in the GC-impaired CXCR4 expression in EPCs. As expected, we found the impaired phosphorylation of AKT in EPCs in the presence of GC; this effect was rescued by the adding of PGE2 which showed by the upregulation of pAKT (Figure 5B). In order to clarify the role of PGE2/PI3K/AKT pathway in the CXCR4 regulation of GC-treated EPCs, we analyzed the expression of CXCR4 in the present of a PI3K inhibitor. The effect of PI3K inhibitor on the phosphorylation of AKT was confirmed (Figure 5C). The data showed that treatment with PI3K inhibitor abolished the rescued effects of PGE2 on the GC-treated EPCs in which CXCR4 expression was impaired (Figure 5D) indicating that PGE2 reversed the GC-impaired CXCR4 expression in EPCs via the activation of PI3K/AKT pathway.

Taken together, these results demonstrated that GC impaired the EP4/AKT signaling which was involved in the downregulation of CXCR4 expression of EPCs.

### GC downregulated the expression of CXCR4 via the independent impairment of the HIF2α and PGE2 pathways

**Figure 6:**
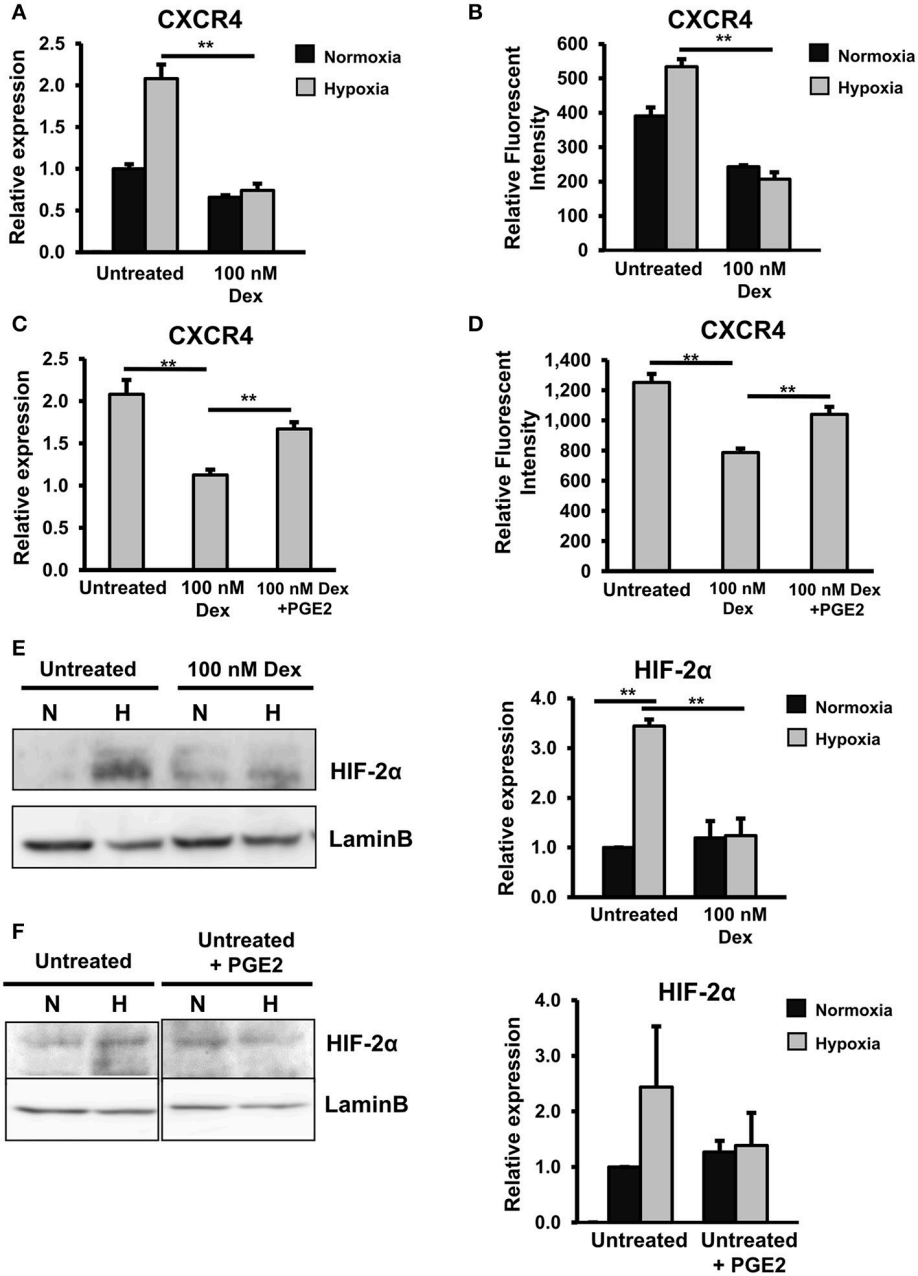
GCs downregulated the expression of CXCR4 via the independent impairment of the HIF2α and PGE2 pathways. **(A)** The relative mRNA expression of CXCR4 in untreated and GC-treated EPCs under normoxic and hypoxic conditions. **(B)** The relative protein expression of CXCR4 in untreated and GC-treated EPCs under normoxic and hypoxic conditions. **(C)** The relative mRNA expression of CXCR4 in untreated EPCs, GC-treated EPCs, and GC-treated EPCs in the presence of PGE2 under hypoxic conditions. **(D)** The relative protein expression of CXCR4 in untreated EPCs, GC-treated EPCs, and GC-treated EPCs in the presence of PGE2 under hypoxic conditions. **(E)** The expression of HIF2α protein in untreated and GC-treated EPCs under normoxic and hypoxic conditions. **(F)** The expression of HIF2α protein in untreated EPCs in the presence of PGE2 under normoxic and hypoxic conditions. Dexamethasone (100 nM, 24-h treatment) was used as a GC. Untreated: untreated EPCs, 100 nM: GC-treated EPCs, 100 nM + PGE2: GC-treated EPCs in the presence of PGE2, Untreated + PGE2: untreated EPCs in the presence of PGE2. The data represent the mean ± SD. *n* = 3, **P < 0.01. The experiment was repeated triplicate. The charts showed the overall mean data from all experiments.

We previously reported the HIF2α-dependent upregulation of CXCR4 in Alde-low EPCs under hypoxic conditions, which promotes the migration and wound healing function of the cells (13, 19). To examine whether or not GC causes similar negative effects that impair the CXCR4 expression in EPCs under hypoxic conditions, we next analyzed untreated and GC-treated EPCs under hypoxic conditions. As expected, in the control EPCs without treatment of GC, we observed the upregulation of CXCR4 at both the mRNA and protein levels under hypoxic conditions (Figures 6A,B). However, in the GC-treated EPCs, the upregulation of CXCR4 under hypoxic conditions was abrogated (Figures 6A,B). Interestingly, this detrimental effect of GC on the CXCR4 expression was rescued by treatment with PGE2 (Figures 6C,D), indicating the similar effects of GC on EPCs under both normoxic and hypoxic conditions of downregulating the expression of CXCR4 via the impairment of the PGE2 pathway.

Our previous report described the functions of the HIF2α pathway, which plays a key role as an upregulator of the CXCR4 expression in EPCs under hypoxic conditions (19). In order to examine whether or not the HIF2α pathway is involved in the GC-impaired reduction of the CXCR4 expression under hypoxic conditions, we next examined the effects of GC on the expression of HIF2α. Interestingly, we found that GC also impaired the expression of HIF2α under hypoxic conditions (Figure 6E).

COX2/PGE2 was reported to upregulate the expression of HIF2α under hypoxic conditions in hepatocellular carcinoma cells (38). Therefore, we thought that PGE2 might also upregulate the expression of HIF2α in EPCs under hypoxic conditions. In order to test this hypothesis, untreated EPCs were cultured in the presence of PGE2 under hypoxic conditions, and the expression of HIF2α was examined. Unexpectedly, we found that PGE2 showed no effect on the expression of HIF2α (Figure 6F). This suggested that the HIF2α and PGE2 pathways independently regulated the expression of CXCR4 in EPCs under hypoxic conditions.

Taken together, these results indicated that GC exerted similar effects on the CXCR4 expression in EPCs under both normoxic and hypoxic conditions. However, under hypoxic conditions, the GC-impaired CXCR4 expression in EPCs was caused independently by the downregulation of the HIF2α or PGE2 pathway.

## Discussion

**Figure 7:**
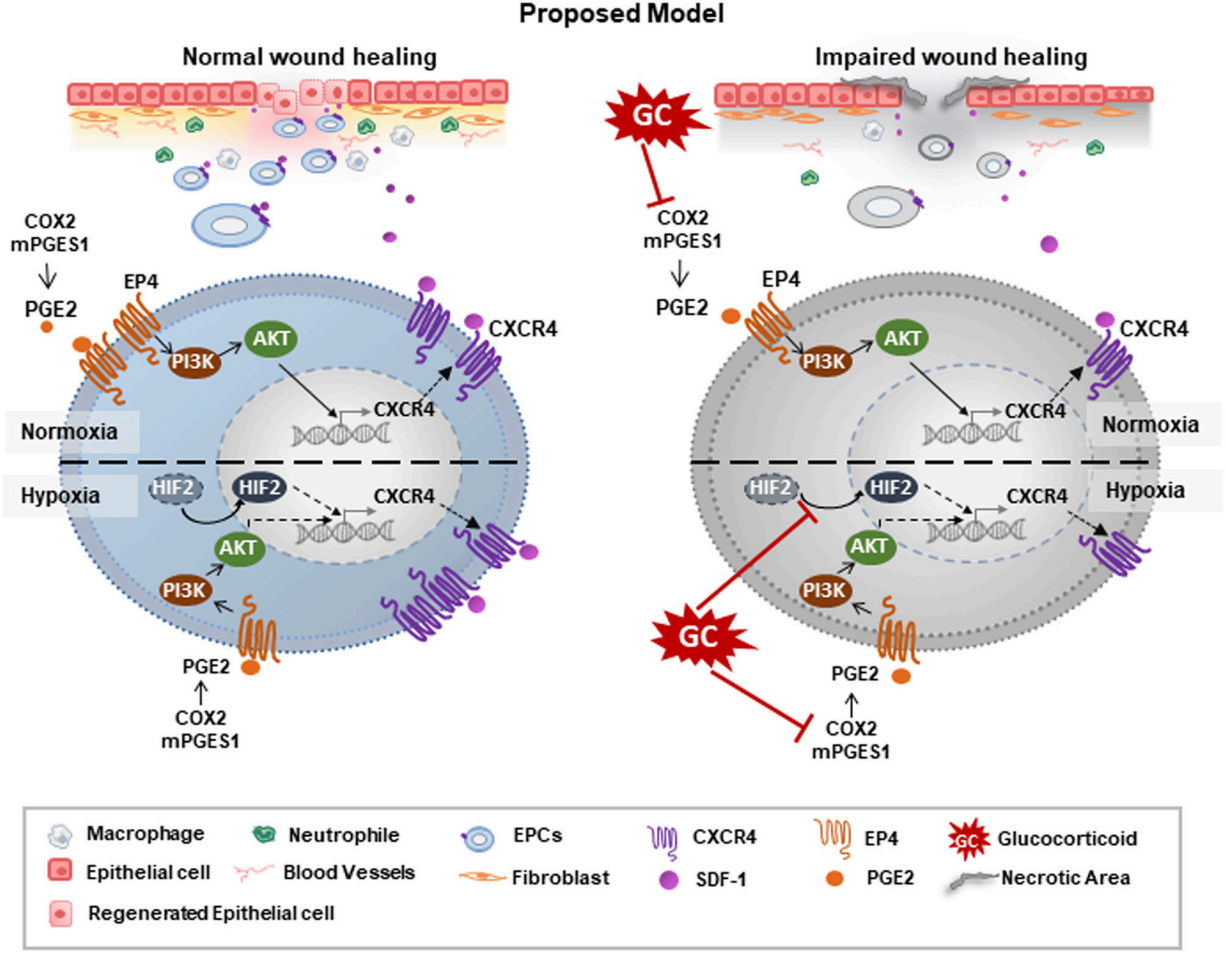
Proposed model: GCs impaired the wound healing functions of EPCs via the impairment of CXCR4 regulated pathways. GCs downregulated the expression of CXCR4 under both normoxic and hypoxic conditions, thereby reducing the migration and wound healing ability of EPCs. Under normoxic condition, GCs impaired the expression of COX2/mPEGS1, thereby suppressing the PGE2/EP4/AKT pathway, which downregulated the CXCR4 expression. Under normoxic conditions, in addition to the PEG2 pathway, GCs independently impaired the HIF2α pathway, consequently decreasing the expression of CXCR4.

In the present study, we showed that GC downregulated the expression of CXCR4, thus abrogating the wound healing ability of EPCs. GC-treated EPCs showed a poor migration ability and dysfunction in the recruitment of inflammatory cells and neovascularization. Of note, the production of PGE2 was associated with the detrimental effects of GCs on EPCs. GCs impaired the expression of COX2 and mPGES1, which are the main enzymes of PGE2 synthesis. Treatment with PGE2 upregulated the expression of EP4 receptors and activated the PI3K/AKT signaling pathways, thereby reversing the impaired effects of GCs on the expression of CXCR4 in EPCs. Importantly, our data demonstrated that, GCs similarly impaired the expression of CXCR4 under hypoxic conditions as under normoxic conditions, as proven by the impairment of PGE2 and another pathway related to HIF2α. Of note, PGE2 and HIF2α acted independently in the regulation of CXCR4 in EPCs under hypoxic conditions (Figure 7, proposed model).

Although no significant influence on the EPCs was observed under microscopic observation, GC treatment decreased the proliferation ability of EPCs. GCs are known to exert anti-proliferative activities against various cells, including neural cells, osteoblasts, osteosarcoma cells, hepatoma cells, and lung, ovarian, and prostate cancer cells (39–41). It has been reported that GC inhibits cell proliferation by arresting the cell cycle via several molecular mechanisms, including the upregulation of cyclin-dependent kinase inhibitors (CKIs), such as p21, that inactivate the cyclinD/Ckd4 complex, leading to G1/G0 arrest (42, 43), or affects other regulators, such as cyclinD1 and c-myc, which induce arrest in the G1 phase (44, 45). In addition, although in our present study, GC treatment showed no apoptosis induction in EPCs after 24-h treatment, a further time-course study is necessary to examine the effects of GC to induce EPCs apoptosis at later time points.

Additionally to the impaired-proliferation, GC treatment downregulated the expression of wound healing genes in EPCs, including CCL2 and CXCR4. CCL2 is the proinflammatory chemokine which is responsible for the inflammatory cell recruitment (25). It is reported that CCL2 promotes wound healing in diabetic mice by inducing macrophage (26). The downregulation of CCL2 in GC-treated EPCs might be involved in the low recruitment of CD45 and Mac1-possitive cells to the wound sites on the third day after transplantation of GC-treated EPCs in the mouse model. The SDF-1/CXCR4 cascade is a major cascade underlying the mobilization of EPCs (46). We previously reported on the GC-impaired wound healing function of MSCs by the suppression of SDF-1 (5). In the present study, although no significant effects on the expression of SDF-1 were noted, GC still adversely affected the wound healing function of EPCs via interference with the regulation of CXCR4. CXCR4 is a G-protein coupled receptor that is highly expressed on EPCs (47). In injured tissues, the surrounding cells secrete SDF-1, which recruits the CXCR4-expressing cells to the wound sites and play their functions in wound healing (48). A study in coronary artery disease patients showed that the dysregulation of CXCR4 signaling reduced the migratory capacity of EPCs in these patients compared to healthy subjects (49). In our study, we found that, under GC treatment, the downregulation of CXCR4, together with the contribution of impaired proliferation, led to a reduced *in vitro* wound healing ability of EPCs in the migration scratch assay. These impaired effects might be involved in the low homing ability of EPCs to the injured tissues in the mouse flap model. These findings suggested the need for further studies on the CXCR4 regulatory effects and functions of EPCs derived from patients receiving GC therapy.

The present findings raised questions about how GCs reduce the CXCR4 expression in EPCs. PGE2 has been proven to induce the expression of CXCR4 in several cells, such as myeloid-derived suppressor cells and microvascular endothelial cells (31, 50). In addition, the inhibitory effects of GCs on prostaglandin synthesis have been well-demonstrated in various cell types and tissues by the mediation of multiple pathways (51, 52). Therefore, we hypothesized that GCs impaired the expression of CXCR4 in EPCs through activity against prostaglandin synthesis. Indeed, our data demonstrated that GCs interfered with the production of PGE2 by the impairment of two enzymes: COX2 and mPGES1. Of note, treatment with PGE2 rescued the expression of CXCR4 in the GC-treated EPCs, which confirmed our hypothesis.

PGE2 is a potent upstream mediator of several genes through interaction with its receptors (EPs), including EP1, EP2, EP3, and EP4, thereby recruiting the transcription factors and modulating the target gene expression (37). Several reports have described the role of PGE2-EPs signaling in the SDF-1-CXCR4 chemokine system (53, 54). EP3 or EP4 knockout suppressed SDF-1 and CXCR4-positive stromal cells in mice (53). PGE2 promotes the homing ability of CD34-positive cells through EP2 and EP4 (54). However, thus far, how GC affects the PGE2 receptors and its effects on the regulation of CXCR4 in EPCs have been unclear. We found that GC impaired the expression of EP4 but not EP1, EP2, or EP3 in EPCs which suggested the involvement of EP4 receptor in this pathway. In addition, previous reports showed that only EP4 regulates the migration of numerous cells (55–57). For instance, EP4 regulates the migration of dendritic cells in mice via selective action on PI3K (55). In addition EP4 is also involved in breast cancer cell migration during tumor invasion (56) and enhances the migration of rat smooth muscle cells (57).

Hypoxia plays a crucial role in modulating the functions of many types of cells via the activation of HIF (58). Hypoxic preconditioning promoted the survival, differentiation, and function of EPCs for the preservation of the left ventricle in acute myocardial ischemia mice (59). In addition, hypoxic treatment upregulated the expression of CXCR4 in EPCs, thereby enhancing the migration of the cells (60). We therefore expected that hypoxic treatment might reverse the negative effects of GCs on the impaired expression of CXCR4. However, our data showed that GC reduced the expression of CXCR4 in EPCs under not only normoxic but also hypoxic conditions, although this impairment was able to be rescued by PGE2. We previously showed that the CXCR4 expression is directly regulated by HIF2α under hypoxic conditions in Alde-low EPCs (19). In our current study, we found that the expression of HIF2α was also downregulated by GC treatment under hypoxic conditions, highlighting another CXCR4-regulated pathway impaired by GC. Previous report described the role of the COX2/PGE2 pathway in the upregulation of HIF2α in carcinoma cells (38); however, in our present study, PGE2 exerted no significant effects on the expression of HIF2α in EPCs, suggesting the cell-specific regulation of HIF2α would exist. In addition, our data indicated the different mechanisms underlying the regulation of CXCR4 under normoxic and hypoxic conditions. Under hypoxic conditions, PGE2 and HIF2α operate as independent pathways that are impaired by GC, consequently suppressing the expression of CXCR4 in EPCs.

Under trauma conditions, peripheral tissues—through myofibroblasts, epithelial cells, and keratinocytes—show an increased production of EPC-mobilizing factors, like VEGF, G-CSF, bFGF, PDGF, and most importantly SDF1, as a potent chemoattractant of EPCs (61). MSCs are considered to contribute to the vascular niche development by providing growth factors (62). SDF-1 production by resident MSCs might therefore encourage the homing ability of EPCs to ischemic sites. A previous report found that the level of SDF-1 protein in the serum of GC-treated patients was significantly decreased compared with untreated patients (63). Because the biologic effects of chemokines are mediated by their corresponding receptors, the interplay between SDF-1 and CXCR4 may provide another way to regulate the distribution of circulating EPCs (64). Similar to our previous finding that GCs reduced SDF-1 production in MSCs (5), the present study showed that GCs also reduced the CXCR4 expression in EPCs. These two discoveries thoroughly show why GC-treated patients have a reduced number of circulating EPCs and a consequently impaired angiogenic ability that leads to dreadful outcomes, like avascular necrotic femoral head (ANFH).

Synthetic GCs have been developed to help treat many different conditions, such as autoimmune disorders, allergies and asthma, cancer, and surgery (24). Despite the multi-functions, GCs causes various side effects including chronic wound (65). It is reported that patients who receive GC treatment for 30 days prior to wounding or operation have a 2-fold increase in wound infection, three times increase in wound dehiscence, and four times increased mortality compared to those who not get the GC treatment (66). In addition, rheumatoid arthritis patients who receive long term GC treatment and surgery have the high risk of delayed wound healing (67). Therefore, it can be implied that the patients with GC-induced chronic wound have become the target of EPC therapy which helps to accelerate wound healing. Our study indicated the negative influences of GC on the wound healing ability of EPCs by the impairment of PGE2/CXCR4, which suggested a strategy to improve the function of EPCs. As autologous cell sources are preferred, the intervention of PGE2 or CXCR4 to improve wound healing ability of GC-treated patients-derived EPCs might be useful before the application to clinical settings.

Previous studies showed that glucocorticoid treatment impairs the corneal neovascularization in mice and rabbit which implied the possibility of abnormal vascularization process, including the dysfunction of EPCs and ECs (68, 69). In addition, EPCs from mice with aldosterone treatment showed the impaired differentiation ability to ECs and migration ability. These studies suggested the *in vivo* negative effects of GCs on EPCs functions (70). However, up to now, the influences of GCs on wound healing ability of EPCs have not yet reported. Therefore, our present study focused on the effects of *in vitro* treated GC on EPCs to provide an idea of how GCs influence EPCs wound healing functions. Further studies related to *in vivo* effects of GCs on EPCs using GC-induced mouse model and GC-treated patients derived EPCs are required to clarify the regulatory ability of GCs on EPCs.

In summary, our study demonstrated that GCs suppress the migration ability and wound healing function of EPCs by the downregulation of CXCR4 under both normoxic and hypoxic conditions. Under normoxic conditions, the impairment of prostaglandin synthases COX2 and mPEGS1 and the prostaglandin receptor EP4 are involved in the detrimental effects on GCs on EPCs. Treatment with PGE2 upregulated the expression of EP4 and consequently activated the PI3K/AKT pathway, which might be involved in rescuing the GC-impaired CXCR4 expression in EPCs. Under hypoxic conditions, in addition to impairment of the PGE2 pathway, GCs exerted similarly detrimental effects on the HIF2α pathway that independently downregulated the expression of CXCR4 in EPCs. Further studies should be performed to carefully assess wound healing functions of EPCs derived from patients who have been receiving long-term treatment with GCs.

## Author contributions

EC, TK, and VK contributed to the study concept, design and access to all the data, data analysis and interpretation, and writing of the manuscript. EC, VK, and OO contributed to the writing and editing of the manuscript. KM, KT, and HH provided samples, technical and laboratory support for the research. TY contributed to the study concept, design, and technical support. OO contributed to the study concept and design, editing of the manuscript and final approval.

### Conflict of interest statement

The authors declare that the research was conducted in the absence of any commercial or financial relationships that could be construed as a potential conflict of interest.
